# Early versus late parenteral nutrition in term and late preterm infants: study protocol for a randomised controlled trial

**DOI:** 10.1186/s12887-022-03569-8

**Published:** 2022-08-30

**Authors:** Kwi Moon, Elizabeth Mckinnon, Kevin Croft, Delia Hendrie, Sanjay Patole, Karen Simmer, Shripada Rao

**Affiliations:** 1 Pharmacy Department, Perth Children’s Hospital, 15 Hospital Ave, Nedlands, WA 6009 Australia; 2 Medical School, The University of Western Australia, Perth, WA Australia; 3 Telethon Kids Institute, Nedlands, WA Australia; 4 School of Biomedical Sciences, The University of Western Australia, Perth, WA Australia; 5 School of Public Health Sciences, Curtin University, Perth, WA Australia; 6 Department of Neonatology, King Edward Memorial Hospital, Subiaco, WA Australia; 7 Department of Neonatology, Perth Children’s Hospital, Nedlands, WA Australia

**Keywords:** Parenteral nutrition, Amino acids, Lipids, F_2_-isoprostanes, Plasma fatty acids, Red blood cell fatty acids, Infant, Neonate, Randomised controlled trial

## Abstract

**Background:**

Despite the wide use of parenteral nutrition (PN) in neonatal intensive care units (NICU), there is limited evidence regarding the optimal time to commence PN in term and late preterm infants. The recommendations from the recently published ESPGHAN/ESPEN/ESPR/CPEN and NICE guidelines are substantially different in this area, and surveys have reported variations in clinical practice. The aim of this randomised controlled trial (RCT) is to evaluate the benefits and risks of early versus late PN in term and late preterm infants.

**Methods/design:**

This study is a single-centre, non-blinded RCT in the NICU of Perth Children’s Hospital, Western Australia.A total of 60 infants born ≥34 weeks of gestation who have a high likelihood of intolerance to enteral nutrition (EN) for at least 3-5 days will be randomised to early (day 1 or day 2 of admission) or late commencement (day 6 of admission) of PN after informed parental consent. In both groups, EN will be commenced as early as clinically feasible. Primary outcomes are plasma phenylalanine and plasma F_2_-isoprostane levels on Day 4 and Day 8 of admission. Secondary outcomes are total and individual plasma amino acid profiles, plasma and red blood cell fatty acid profiles, in-hospital all-cause mortality, hospital-acquired infections, length of hospital/NICU stay, z scores and changes in z scores at discharge for weight, height and head circumference, time to full EN, duration of respiratory (mechanical, non-invasive) support, duration of inotropic support, the incidence of hyper and hypoglycaemia, incidence of metabolic acidosis, liver function, blood urea nitrogen, and C-reactive protein (CRP).

**Discussion:**

This RCT will examine the effects of early versus late PN in term and late preterm infants by comparing key biochemical and clinical outcomes and has the potential to identify underlying pathways for beneficial or harmful effects related to the timing of commencement of PN in such infants.

**Trial registration:**

ANZCTR; ACTRN12620000324910 (3rd March 2020)

## Background

Term and late preterm infants (≥34 weeks of gestation) may suffer from conditions that predispose them to critical illness needing admission to Neonatal Intensive Care Units (NICU). These include persistent pulmonary hypertension of the newborn (PPHN) [1], meconium aspiration syndrome [2], hypoxic-ischaemic encephalopathy (HIE) [3], sepsis [4], critical congenital heart disease [5], congenital diaphragmatic hernia [6], and other major surgical conditions [7].

During periods of acute illness, the provision of enteral nutrition (EN) may be unachievable, which necessitates the use of parenteral nutrition (PN). PN involves the administration of glucose, amino acids, lipids and various micronutrients and is usually indicated in infants in whom EN is contraindicated or delivers insufficient protein and energy requirements [8].

Despite the wide use of PN in NICU, there is limited evidence regarding the optimal time to commence PN in term and late preterm infants [9, 10]. Evidence regarding early versus late PN in term and late preterm infants is limited to the subgroup analysis of 209 term infants [11] from the PEPaNIC trial, a multi-centre RCT of critically ill children (*N* = 1440; birth to 17 years) [12]. In that study, the early PN group received PN within 24 h of admission and late PN group, after 7 days. They reported that, late PN was associated with earlier discharge from the ICU but increased the risk of hypoglycaemia in neonates [11]. Due to the various study limitations and concerns about hypoglycaemia, experts do not recommend withholding PN for 7 days as standard practice [10, 13]. Furthermore, our Cochrane review, which only identified data from the PEPaNIC trial, also concluded that the quality of the evidence was low, highlighting the need for further RCTs [9].

A recent retrospective cohort study found that preterm infants (born between 30^+ 0^ and 32^+ 6^ weeks’ gestation) who were commenced PN within the first 7 days of life had a higher rate of survival but higher rates of necrotising enterocolitis, bronchopulmonary dysplasia, late-onset sepsis and the need for surgical procedures [14].

The recommendations from the recently published ESPGHAN/ESPEN/ESPR/CPEN and NICE guidelines are substantially different in this area. The NICE guidelines recommend the commencement of PN within 72 h after birth if no progress is made with EN in term and late preterm infants [15]. On the other hand, the ESPGHAN/ESPEN/ESPR/CPEN guidelines recommend clinicians to consider withholding *parenteral amino acids* for 1 week in critically ill term infants; they do not make a recommendation on the timing of *parenteral lipids* [16]. The surveys have reported variations in clinical practice, demonstrating the uncertainties in this area [17–19].

Amino acids are major and essential constituents of PN; they are precursors of protein synthesis and signalling molecules that regulate energy metabolism, cell proliferation and survival [20]. Early parenteral amino acids may have beneficial effects by preventing catabolism, reducing the risk of hyperglycaemia and enhancing the secretion of insulin-like growth factor and may lead to improved growth and neurodevelopmental outcomes [21–27]. However, there are concerns about the harmful effects of parenteral amino acids such as hyperammonaemia, azotaemia, metabolic acidosis, free radical injury, and slower growth of head circumference and metabolic syndromes later in life [24, 28–31].

High levels of plasma phenylalanine and other amino acids have been observed in critically ill term and late preterm infants [32–38], suggesting metabolic derangements of certain amino acids during critical illness. If such infants are administered parenteral amino acids early during their critical illness, plasma levels may reach very high levels, leading to potential harm. In the cohort of critically ill children undergoing cardiac surgery, non-surviving children had higher plasma amino acid levels compared to survivors [39]. In an RCT of extremely low birth weight infants, those receiving early and higher parenteral amino acids had higher plasma amino acid levels [40]. The early and high amino acid group had a lower mental developmental index (MDI) at 18 months and poor overall growth at 18- 24 months [41]. Authors reported that higher levels of phenylalanine, isoleucine, valine, and leucine, were associated with poor growth and low MDI and/or Psychomotor Developmental Index scores [41]. More recently, in the subgroup analysis of 209 term infants of the PEPaNIC trial, higher amino acid doses were associated with prolonged ICU stay and duration of mechanical ventilation [11]. The authors postulated that the harm associated with parenteral amino acids might be caused by the suppression of autophagy, the amino acid load being above the metabolic capacity of the liver and kidney or suboptimal composition of amino acid formulations [11].

Intravenous lipid emulsions are another important component of PN. They provide a concentrated source of energy, essential fatty acids (EFA) and long-chain polyunsaturated fatty acids (LCPUFA). EFA deficiency can be prevented by giving as little as 0.5-1 g/kg/day lipid emulsions [42–44]. Early parenteral lipids can prevent EFA deficiency, increase LCPUFA levels and have the potential to improve neurodevelopmental outcomes. On the other hand, lipid emulsions may cause harmful effects by exacerbating free radical activity [45, 46], increasing the risk of sepsis [47, 48], worsening respiratory function by decreasing arterial oxygenation [49–51], increasing the risk of kernicterus in infants with hyperbilirubinaemia due to free fatty acids displacing bilirubin from albumin binding sites [52] and may worsen pulmonary hypertension [53].

Intake of PUFA from parenteral lipids and various components of PN is associated with the production of free radicals resulting in an increased risk of membrane peroxidation and may worsen the oxidative stress in critically ill infants [31, 54–61]. Basu et al. found high malondialdehyde levels (a marker of free radical activity) in critically ill as well as stable infants receiving PN, but the levels were even higher in critically ill infants [45]. Given that critically ill infants in the NICU (e.g. HIE, PPHN, necrotising enterocolitis, congenital heart disease and sepsis) are already under oxidative stress [62–67], early commencement of PN may worsen it.

Furthermore, evidence is accumulating that aggressive nutrition during the early phase of critical illness may be associated with enhanced oxidative stress and attenuation of autophagy [68]. In mechanically ventilated adults with systemic inflammatory response syndrome, oxidative stress (measured by total plasma F_2_-isoprostanes) was significantly higher in participants in the second or third versus the first tertile of real-time energy flow rate [69]. Hence, the authors of that study postulated that high energy induced oxidative stress may be a potential mechanism of harm associated with early PN use in adults [69].

This RCT will examine the effects of early versus late PN in term and late preterm infants by comparing key biochemical and clinical outcomes and has the potential to identify underlying pathways for beneficial or harmful effects related to the timing of commencement of PN in such infants.

## Methods/design

### Aim and hypothesis

Our current RCT aims to evaluate the benefits and risks of early versus late PN in term and late preterm infants. We hypothesise that late commencement of PN will achieve lower (and more physiological) levels of plasma phenylalanine and plasma F_2_-isoprostanes on Day 4 and Day 8 compared to early commencement of PN in term and late preterm infants.

### Objectives

#### Primary objective

The key objective of this RCT is to compare the effect of early vs late commencement of parenteral nutrition on plasma phenylalanine and F_2_-isoprostane levels on Day 4 and Day 8 in term and late preterm infants.

The *secondary objectives* are to:

- Characterise the overall plasma amino acid and fatty acid profiles (red blood cell (RBC) and plasma) of infants receiving early versus late PN
- Describe the clinical outcomes of early versus late PN in study infants
- Explore associations of plasma amino acid and fatty acid profiles (RBC and plasma) and plasma F_2_-isoprostane levels with clinical and other biochemical outcomes
- Perform cost analysis of early versus late PN from an Australian public hospital perspective

### Study design and setting

**Fig. 1:**
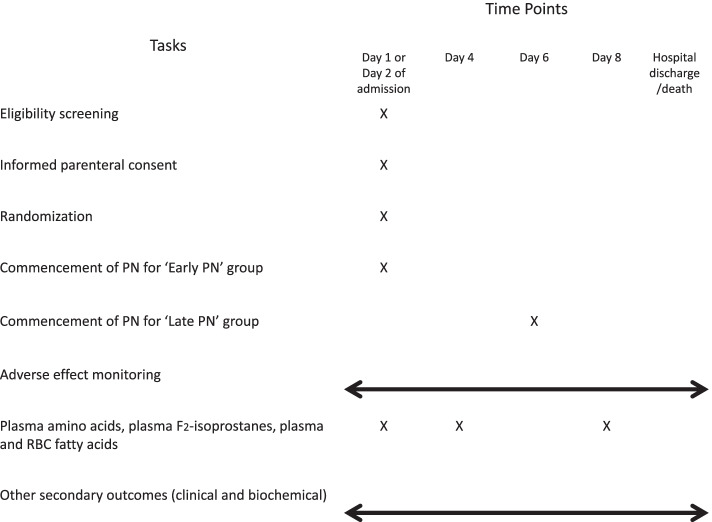
Study Schedule

A prospective, single-centre, non-blinded RCT in the neonatal intensive care unit (NICU) of Perth Children’s Hospital (PCH), Child and Adolescent Health Service (CAHS), Perth, Australia. PCH-NICU is a 30-bed tertiary referral centre that provides intensive and high dependency care for critically ill neonates. Over 800 infants are admitted to our NICU each year and all infants are out born. The study schedule is shown in Fig. 1.

### Study population

#### Inclusion criteria

1. Term or late preterm infants born ≥34 weeks of gestation and ≤ 28 days old, admitted to NICU from another hospital who have a high likelihood of being unable to tolerate enteral feeds for at least 3 to 5 days-This definition and judgement was based on our retrospective audit of 124 late preterm and term infants (currently in peer review), which found that conditions such as gastroschisis, tracheoesophageal fistula, meconium aspiration syndrome, PPHN and HIE wereunlikely to tolerate full enteral feeds by five days of admission.
2. Informed parental/guardian consent

#### Exclusion criteria

1. Infants who received PN at a referring hospital
2. Infants with a suspected inborn error of metabolism
3. Infants who previously established full enteral feeding or has had more than 50% of total fluids as EN for more than 12 h since birth
4. Infants admitted from home

### Recruitment

A parent/guardian of an eligible infant will be approached by the Coordinating Principal Investigator (CPI) or her delegate and invited to participate in this study. The CPI will accredit the delegates to take parental consents. Informed parental consent to trial participation is required by Day 2 of admission so that the trial intervention and data collection can be initiated by Day 2 of admission.

### Interventions

With Day 1 defined as the day of admission if admitted before 12.00 pm, and the following day if admitted after 12.00 pm, participants will be allocated to one of the two types of interventions, either ‘Early PN’ or ‘Late PN’. Day 1 of admission is not always Day 1 of life as participants will be out born.

#### Early PN (standard care or control group)

Infants randomised to ‘Early PN’ will be commenced PN on Day 1 or Day 2 of admission to the NICU.

#### Late PN (intervention group)

Infants randomised to ‘Late PN’ will commence PN only after the completion of Day 5 of NICU admission. If an infant randomised to ‘late PN’ does not require PN after Day 5, the infant will still be included as being in the ‘Late PN’ group and analysed as such.

#### Justifications for the definition of ‘late PN’ (intervention group)

Early versus late parenteral nutrition (PN) trials in adults and children (birth to 17 years) defined early PN as “within 24 h of admission” and late PN as “after 7 days of admission” [12]. As highlighted by Mehta et al., they are two extreme timings and practised only in a very few units [13]. Hence such a study design is unlikely to be feasible in term and late preterm infants.

We conducted a survey (September 2019) to find the current practice and to seek the opinions of clinicians (neonatologists and paediatric intensivists) in Australia and New Zealand [70]. We found that 43% of respondents (32/74) thought that commencing parenteral amino acids “within 24 h of admission” is beneficial, while 32% (24/74) felt that late commencement “after 72 h” is optimal. None of the respondents felt “after 7 days of admission” was the optimal time to commence parenteral amino acids. Similarly, 41% (31/75) thought that commencement of parenteral lipids “within 24 h of admission” is optimal, whereas 35% (26/74) felt late commencement “after 72 h” to be beneficial. Only one respondent thought “after 7 days of admission” was an optimal time to commence lipids [70].

The Cochrane review (Moon et al), defined late PN as “after 72 h of admission” [9].

After carefully considering the above information, our trial steering committee decided that ‘late PN’ should be > 72 h but < 7 days of admission. We settled on day 6 as the definition for late PN to maximise the time difference between the early and late PN groups, while at the same time ensuring feasibility for future multicentre RCTs.

### PN

PN will be defined as the administration of any amount of intravenous amino acids and/or lipid emulsion. Administration of PN will be either via peripheral intravenous cannula or central venous access device. Prescription of PN (including the type of PN, dosage and rate of administration) will be determined by the treating clinician daily, depending on the infant’s clinical progress and according to the CAHS Neonatology Guidelines (“Nutrition: Volume and Nutritional Requirements” and “Parenteral Nutrition”). Usually, on day 1 of admission, 60 to 80 mL/kg/day of parenteral amino acids solution (2.3% amino acids and 12% glucose) and 6 mL/kg/day of lipid emulsion 17% are administered to infants to provide 1.4-1.8 g/kg/day of amino acids and 1 g/kg/day of lipids respectively. When an infant receives 140 mL/kg/day of parenteral amino acids and 18 mL/kg/day of lipid emulsions, amino acid and lipid intake of 3.2 g/kg/day and 3 g/kg/day are reached, respectively.

All PN formulations will be prepared by the Pharmacy Department at PCH or an external compounding provider. Standard PNs used in the NICU contains 2.3 g or 3 g/100 mL of amino acids and 12 g or 8 g/100 mL of glucose with standard amounts of electrolytes, trace elements and heparin. Lipid emulsions 20% (SMOF®, Fresenius, Uppsala, Sweden) consisting of soya oil (30%), medium-chain triglyceride (30%), refined olive oil (25%) and fish oil (15%) will be used as the source of parenteral lipids. Both fat-soluble (Soluvit N®, Fresenius, Uppsala, Sweden) and water-soluble vitamins (Vitalipid N®, Fresenius, Uppsala, Sweden) will be added to the lipid emulsion in the Pharmacy, resulting in lipid emulsion 17%. Each 6 mL of lipid 17% contains 1 g fat.

Infants randomised to the ‘early PN’ group will receive PN formulation as prescribed by the treating clinician on Day 1 or Day 2 of admission to NICU.

Infants randomised to the ‘late PN’ group will receive glucose and sodium chloride solutions as per infant’s needs up to Day 5 and then commence PN from Day 6 of admission to NICU.

PN will be prescribed and administered according to the CAHS Neonatology Guidelines: Volume and Nutritional Requirements. Administration of PN and glucose, sodium and other electrolytes will be titrated according to routine biochemical results (examples: blood glucose, serum sodium, potassium, magnesium, calcium) by the treating clinician.

### EN

EN will be commenced as early as clinically feasible in both intervention groups per the CAHS Neonatology Guidelines: Enteral feeding- Initiation and progression. When EN provides 80-100% of target nutrition intake (150 mL/kg/day) or clinically appropriate, PN will be stopped by the treating clinician. EN of choice will be mother’s own milk. Standard formula milk will be used if mother’s milk supply is inadequate or if it is mother’s preference. Extensively hydrolysed formulas will be used occasionally only if clinically indicated.

### Assignment of interventions

#### Random sequence generation

Group assignment will be allocated by the Clinical Trial Pharmacist at PCH using a computer-generated randomisation sequence in a 1:1 ratio in block sizes of 2 and 4 and stratified according to the primary diagnostic category (medical or surgical) to minimise selection bias.

#### Allocation concealment

Allocation concealment will be achieved by using opaque sealed envelopes. Once consent is provided, the CPI or delegate will open the next opaque sealed envelope to allocate the infant to the ‘Early PN’ or ‘Late PN’ group.

#### Blinding

Blinding of families, healthcare providers and study investigators to group allocation is not possible for this intervention. However, the laboratory personnel responsible for plasma amino acids, F_2_-isoprostanes and plasma/RBC fatty acids and the trial biostatistician will be blinded to allocations.

### Study outcomes

#### Primary endpoints

The two primary endpoints of this RCT are levels of (a) plasma phenylalanine (μmol/L) and (b) plasma F_2_-isoprostane (pg/mL) on Day 4 and Day 8 of admission.

##### Justifications for plasma phenylalanine as the primary endpoint

We hypothesise that early parenteral amino acids may not be tolerated in critically ill infants in the acute phase of illness and may lead to high plasma phenylalanine concentrations.

There are many reports describing abnormal plasma amino acid profiles (especially phenylalanine) in critically ill neonates [32, 33, 36–38, 71]. High phenylalanine levels have been reported in infants with PPHN, peritonitis, HIE, sepsis and congenital heart defects (post-operative) [32, 33, 36–38, 71]. Suggested mechanisms for such high levels are impaired phenylalanine hydroxylase (PAH) activity due to oxidative stress [36], decreased metabolism in the liver [33], increased muscle breakdown [37], reduced incorporation of phenylalanine into muscle protein [37], altered rate of hepatic conversion of phenylalanine to tyrosine [37]. The relevance of the high phenylalanine levels during critical illness is that it is associated with higher mortality [33, 39], poor growth and neurodevelopmental outcomes in neonates [41]. It is well known that elevated levels in conditions such as phenylketonuria are associated with brain injury [72]. Hence, we have chosen phenylalanine as a primary endpoint and also to evaluate full amino acid profile in our study infants.

##### Justifications for plasma F_2_-isoprostanes as the primary endpoint

Another potential mechanism through which early PN can cause harm is through worsening of oxidative stress. Oxidative stress is a major characteristic of various diseases in humans. In the context of critically ill term and late preterm infants, sources of oxidative stress are birth trauma, reperfusion injury after hypoxia/ischaemia, oxygen therapy, acidosis, phototherapy, mechanical ventilation, infection, and inflammation [73–75]. Oxidative stress is present in conditions that predispose to critical illness in term and late preterm infants. The examples are congenital heart disease [62], PPHN [63], HIE [64], NEC [65] and sepsis [66, 67]. In the presence of oxygen, nutrients such as lipids, amino acids and vitamins become potent electric donors that promote the generation of peroxides [31]. Polyunsaturated fatty acids present in lipid emulsions are susceptible to oxidation [54, 57]. Furthermore, various studies have shown that oxidation of amino acid solutions, vitamins and trace elements can also result in increased oxidative stress [45, 58–61]. Hence, early intake of PN may worsen the oxidative stress in critically ill infants.

It is well known that F_2_-isoprostanes are sensitive markers of oxidative stress. They are prostaglandin like substances that are formed in vivo by free radical-induced peroxidation of arachidonic acids [76]. F_2_-isoprostanes are a. chemically stable, b. specific products of peroxidation, c. formed in vivo, d. present in detectable amounts in all normal tissues and biological fluids and e.unaffected by lipid contents in the diet [77]. All these characteristics make them a suitable candidate as a biomarker of oxidative stress. In addition, recent studies have found that increased F_2_-isoprostane levels are associated with poor neurodevelopment and neurological outcomes in infants [62, 78, 79] Hence, we have chosen plasma F_2_-isoprostanes as one of the primary endpoint in our RCT.

#### Secondary safety endpoints

- Incidence of hypoglycaemia [blood glucose < 2.6 mmol/L] (%)
- Incidence of hypoglycaemia resistant to parenteral glucose administration (%)
- In-hospital mortality [defined as death from any cause during the hospital stay] (%)
- Neonatal mortality [defined as death in the first 28 days from birth since birth from any cause] (%)

#### Secondary efficacy endpoints

- Total and individual plasma amino acids on Day 4 and Day 8 of admission [Plasma amino acid profiles in healthy term breastfed infants have been studied, describing the optimal plasma amino acid pattern for growth and development [80, 81]. These data will be used as reference values] (μmol/L)
- Plasma and RBC fatty acid profiles on Day 4 and Day 8 of admission [Plasma and RBC fatty acid compositions of healthy term breastfed infants have been reported [82–86]. These data will be used as reference values] (% total)

#### Secondary clinical endpoints

- Duration of NICU stay (days)
- Duration of hospital stay (days)
- Duration of respiratory support [defined as mechanical ventilation via endotracheal tube OR non-invasive ventilation (NIPPV) or continuous positive airway pressure (CPAP, or humidified high-flow nasal prong (HFNP) therapy] (days)
- Duration of mechanical ventilation via an endotracheal tube (days)
- Weight, length and head circumference z scores at the time of discharge from the hospital
- Changes in z scores for weight, length and head circumference from baseline todischarge
- Time to full EN (days). Full EN will be defined as enteral nutrition intake of ≥150 mL/kg/day.
- Number of hypoglycaemia episodes [blood glucose < 2.6 mmol/L] (counts)
- Number of hyperglycaemia episodes [blood glucose> 8.3 mmol/L] (counts)
- Incidence of hospital-acquired infections [defined as positive growth on blood culture or cerebrospinal fluid or sterile urine or wound swabs drawn 48 h after admission to NICU] (%)
- Duration of antibiotic treatment (days)
- Duration of ionotropic support (hours)
- Highest blood urea nitrogen (BUN) during hospital stay (mmol/L)
- Incidence of metabolic acidosis [pH < 7.25 or base excess <− 5] during hospital stay (%)
- Highest C-reactive protein during hospital stay (mg/L)
- Incidence of liver dysfunction [raised liver function tests including total bilirubin (> 200micromol/L), alkaline phosphatase (> 380 units/L), γ-glutamyltransferase (> 150 units/L), alanine transferase (> 35 units/L)] during hospital stay (%)
- Incidence of cholestasis [serum level of direct bilirubin> 20% of total serum bilirubin or serum level of direct bilirubin > 34 mmol/L] during hospital stay (%)

### Statistical considerations

#### Sample size

We will recruit 60 infants (30 in each arm), which is a feasible number given that the NICU at PCH currently admits approximately 120 infants per year who would meet the eligibility criteria. Assuming the covariate-adjusted within-group standard deviation of F_2_-isoprostane levels in term/late pre-term infants is approximately 25 pg/mL [87], we would therefore have 80% power to detect a difference in early versus late PN levels of 16.2 pg/mL and 90% power to detect a difference of 19.1 pg/mL (α = 0.05). Levels of F_2_-isoprostane at birth have spanned a range of approximately 40 pg/mL across 35-40 weeks gestation [87] indicating the minimal detectable difference is clinically relevant. This study will also have adequate power to detect an increase of approximately 20% in mean phenylalanine levels in infants receiving early PN, noting that mean Day 3 levels in extremely low birth weight infants receiving aggressive nutritional therapy were at least 25% higher than that in infants receiving the standard in the study by Blanco et al. [40]. In the majority of study infants, we will collect blood through existing arterial or central venous lines. In a small number of infants (*n* = 5 to10) in whom such access is not available, we will perform venepuncture to collect the blood samples. To account for the possibility that venepuncture may be unsuccessful, we will recruit an additional three infants.

#### Statistical analyses

Baseline characteristics of study participants will be reported as counts (percentages) for categorical outcomes, and mean (standard deviation) for normally distributed continuous outcomes or median (interquartile range) for skewed continuous outcomes.

The primary ANCOVA analyses will assess differences between the two PN groups in F_2_-isoprostane and log-transformed phenylalanine levels at Days 4 and 8. The analysis will be based on the principle of intention to treat, with the last blood plasma values carried forward for infants discharged prior to the day 8 blood collection. Covariate adjustment will be made for baseline F_2_-isoprostanes/phenylalanine, sex, gestational age, medical/surgical group, and severity of illness scores (Score for Neonatal Acute Physiology with Perinatal Extension-II (SNAPPE-II) [88].

Linear regression will be used for the analysis of the secondary efficacy outcomes (amino acid and fatty acid profiles) and growth outcomes. Trajectories of F_2_-isoprostanes, plasma amino acids and fatty acids will be examined within a mixed-effects regression framework to account for the multiple measures per infant.

Time to full EN, morbidity and respiratory outcomes (duration of hospital/NICU stay, respiratory support/mechanical ventilation) will be analysed as time-to-event data using Cox regression, with data censored at 90 days for non-survivors and survivors not reaching the endpoint within that time. Other clinical outcomes will be reported descriptively.

Appropriate regression analyses will also explore the association of the biochemical markers (F_2_-isoprostane, plasma amino acids and plasma /RBC fatty acids) with the main clinical outcomes.

Regression analyses will assess PN group differences in an unadjusted manner, as well as (jointly) controlled for risk factors including sex, gestational age, medical/surgical group, birth weight z scores and severity of illness scores (SNAPPE-II) [88]. We will investigate independent effects of 1) Medical and surgical groups; 2) Term (≥ 37 weeks’ PMA) and late preterm groups (34 to 36 6/7 weeks’ PMA). Covariate data that can be reasonably judged to be missing at random will be inferred using an appropriate method of multiple imputations.

Summary statistics for main outcome measures will include 95% confidence intervals, and for all endpoints, differences between PN groups will be considered statistically significant whenever the *p*-value is lower than 0.05 without correction for multiple testing.

### Cost analysis

The results of this RCT will be used to perform a cost-consequence analysis from a tertiary Australian public hospital perspective that utilises clinical costing under an Activity Based Funding model. We will estimate and compare direct medical costs and key clinical outcomes associated with early versus late PN. We will obtain clinical costing estimates for each participant in the trial from the CAHS Business Intelligence Unit (CAHS BIU). Like other Health Care Service Providers in WA, CAHS patient-level cost estimates are calculated by leveraging the Power Performance Manager® (PPM). Data files such as general ledger, patient encounter, transfer, diagnosis, procedure, allied health, pathology, radiology, theatre and medical are loaded into the PPM. Costs are allocated to each patient by linking services to an encounter such that volumes of usage are multiplied by corresponding unit prices of each cost category. Categories for direct medical cost include 1) hotel; 2) radiology; 3) medical/surgical supplies; 4) pathology; 5) pharmacy; 6) prostheses; 7) clinical staff (medical, nursing, allied health). As pharmacy costs, including PN cost, are not routinely calculated at a patient level, we will calculate PN and drug cost using micro-costing approach. All other cost categories are allocated at a patient level. Mean costs and 95% CI of differences in per-patient costs between the two groups will be compared for total direct medical costs and selected cost categories. Given the highly skewed distribution, which is typical of cost data, non-parametric cost comparisons will also be made.

### Data collection following recruitment

#### Blood sample collections, processing and storage

The whole blood volume of 1.4 mL will be drawn from the study infants via IV access placed for clinical purpose or by venepuncture in accordance with the CAHS Neonatology Guideline (Blood sampling: venepuncture, peripheral arterial, UAC, UVC and CVC).

After a blood sample is drawn by nursing or medical staff in NICU, the sample will be transferred on ice at 4 °C for centrifugation at 4 °C within 2 h of collection. Plasma will be extracted and transferred to small cryovials (0.2 mL for amino acid analysis, 0.25 mL for F2-isoprostanes, 0.05 mL for fatty acids). Any remaining plasma will be stored in a 4th cryovial. Following extraction of plasma from the whole blood sample, the remaining RBC cell sample will be stored in a 5th cryovial for RBC fatty acids. The samples will be stored at − 80 °C. Subsequently, the processed plasma and RBC samples will be transported to external laboratories for analyses on dry ice in an esky. We will use World Courier or Lab Taxi for deliveries to external laboratories. Each sample stored will be clearly labelled with a unique identifiable code, sample day and volume. If an infant is discharged from NICU prior to Day 8, we plan to collect the third sample on the day of discharge.

#### Plasma phenylalanine and amino acids

The concentrations of amino acids will be determined using pre-column derivatisation amino acid analysis with 6-aminoquinolyl-N-hydroxysuccinimidyl carbamate (AQC; Cohen SA, 1993, 2001) followed by separation of the derivatives and quantification by modified reversed phase ultra-performance binary liquid chromatography (UPLC; Waters Corporation; Milford, Mass. USA) employing a 12-min gradient.

Plasma, stored at − 80 °C will be thawed to room temperature and then diluted 1:1 with an internal standard (Norvaline and α amino butyric acid; Nva/AABA). The solution will be deproteinated by ultrafiltration through a membrane with a 10 kDa nominal molecular weight cut-off filter (Amicon® Ultra Centrifugal Filters, Merk Millipore). The free amino acids in the filtrate will be derivatised using the AccQ-Tag Ultra Derivatization Kit (Waters Corp) following the suppliers recommended procedures. Detection will be at 260 nm (UV). Quantitation of the amino acids will employ the Empower software (Waters Corporation) using a set of prepared standards, with Nva/AABA as the internal standard.

#### Plasma F2-isoprostanes

Plasma total F_2_-isoprostane concentrations will be measured by gas chromatography coupled to electron-capture negative-mode chemical ionisation and mass spectrometry using a modification of the previously reported method [89]. F_2_-isoprostanes in plasma (0.25 ml) will be hydrolysed under N_2_ with 1 M-KOH in methanol for 40 min at 408C. The sample will be acidified to a pH of 4.0 before being subjected to solid-phase chromatography on a pre-washed 200 mg Certify II column (Varian).

After eluting with methanol-water (50:50, v/v) and hexane- ethyl acetate (75:25, v/v), the F_2_-isoprostanes will be eluted with 2 ml ethyl acetate-methanol (90:10), dried and derivatised. F_2_-isoprostanes will be detected by selected ion monitoring using m/z 569 and m/z 573 for endogenous and tetra-deuterated internal standards, respectively.

#### Plasma and RBC fatty acids

Plasma and RBC fatty acids Plasma (0.05 mL) and BF3: Toluene: Methanol (350:300:350; 400ul) in a capped 3 ml glass vial will be vortexed and heated for 45 min at 100 °C. The samples are to be cooled and hexane (250 μl) and high-performance liquid chromatography grade water (250 μl) are added, vortex and centrifuge at 3500 rpm for 3 mins at 4 °C.The hexane fraction (30 μl) is analysed by gas-liquid chromatography. Peaks are identified by comparison with a known standard mixture. Individual fatty acids are calculated as a relative percentage with the evaluated fatty acids set at 100%.

#### Nutrition intake

Total parenteral and enteral nutrition intakes will be recorded daily until discharge. Daily nutrition intake will be calculated based on actual intake. Full enteral nutrition will be defined as 150 mL/kg/day or exclusive breastfeeding. Energy and protein intake from EN will be calculated using CAHS Clinical Guideline (Milk Room: Breast milk fortification and preterm formula).

#### Clinical data

All clinical data such as patient characteristics (e.g. main diagnosis, type of surgery received, inotrope use, antibiotics use, invasive ventilation use, non-invasive ventilation use) and secondary outcomes will be collected daily using medical records.

#### Data management

De-identified study data will be collected by the CPI and entered into a secure web-based application (REDCap®) specifically designed and built to capture, manage, and store health research data, which will be hosted on a secure server at the Telethon Kids Institute (TKI). Data collected will be accessed only by the authorised members of the study team named on the HREC application for the study. Blood samples will be stored securely at -80 °C at the TKI until the samples are transported securely to the laboratories.

### Data and safety monitoring

An independent Data and Safety Monitoring Committee (DSMC) has been established to ensure the safety of the trial participants and to monitor the quality of the study and data collection. The DSMC will comprise a Neonatologist and Senior Pharmacist who are external to PCH and are not involved in the trial. DSMC will review the study data and conduct interim analyses following recruitment of 20 and 40 participants for the key safety outcomes: incidence of hypoglycaemia, hypoglycaemia resistant to parenteral glucose administration, in-hospital mortality, neonatal mortality and any perceived life-threatening event during hospitalisation associated with the intervention.

All adverse events will be reported to the HREC of CAHS and DSMC within one business day of the first awareness of the event.

### Trial termination

The DSMC may make recommendations to stop the trial if there are concerns that either of the study interventions is harmful. We will use an unadjusted chi-square test to compare the key serious adverse events (i.e. in-hospital mortality, neonatal mortality and hypoglycaemia resistant to parenteral glucose administration) between the early and late PN groups. If there is a significant difference between the two groups, the DSMC may consider stopping the trial. Furthermore, given the known rare incidence of death during admission to NICU at PCH (i.e. 15 to 20 cases per year out of approximately 720 admissions), any death during the study will result in a pause of the study and review of death by the DSMC. The DSMC determines whether it is study-related or not before continuing the study and will stop the study if death is deemed to be related to the study.

### Ethics and dissemination

Ethics approval for all aspects of this study has been granted (RGS 0000003537). Deviation from this protocol will occur only with prior approval from HREC. The study will be conducted in accordance with the International Council on Harmonisation Good Clinical Practice guidance documents and the National Health and Medical Research Council National Statement of Ethical conduct in Human research.

The proposed interventions are minimally invasive and have negligible ethical implications for the infants assigned to the intervention groups.

As infants are unable to provide consent, informed consent will be obtained prospectively from the parent/guardian of eligible infants for the participation of their infant in the study. Great care will be taken to maintain the highest standards regarding consent for clinical research.

The CPI or a delegate will provide the parent/guardian with a trial information sheet. Trial information will be discussed in detail with the parent/guardian, and their questions will be answered in full. Once the parent/guardian are happy with the information and agree to enrolment, their signature will be taken on the Human Research Ethics Committee (HREC) approved consent form prior to randomising the infant. Optional consent will be obtained to keep any unused blood samples for future research related to the current research and to contact the parent/guardian at a later date for further research related to the current research.

Participation in this study is voluntary. A parent/guardian can withdraw their infant at any time without giving a reason. No additional information will be collected. We will use the data and samples already collected unless the parent/guardian advises us not to. The withdrawal of participation will not prejudice current or future medical treatments.

The original consent form will be filed in the study data file, the parent/guardian will be given a copy and documentation within the patient notes concerning the type of study the patient is enrolled in will occur.

The trial is registered with Australia and New Zealand Clinical Trials Registry (ACTRN12620000324910). The outcomes of this study will be submitted for publication in a peer-reviewed journal and presentation at scientific meetings. CONSORT checklist [90] will be followed for reporting the results in a journal.

## Discussion

The clinical evidence for the optimal time to commence PN in term and late preterm infants is limited. This RCT aims to examine the benefits and risks of early versus late PN in term and late preterm infants. The study will assess whether different times for commencing PN in these infants has any impact on plasma amino acid, oxidative stress (via F_2_-isoprostanes) and plasma and RBC fatty acid levels. The study will also assess the clinical outcomes of infants across the two groups. Although our study is not powered to find differences in these clinical outcomes, it will be helpful in providing directionality. These results will enable us to understand the newborn infant’s ability to utilise and tolerate PN during their acute illness. The undertaking of exploratory analyses examining the association of the biochemical outcomes to the key clinical outcomes will allow gaining insights into the macronutrients associated with beneficial and harmful clinical effects.

Furthermore, there is no published health economic study that has evaluated the cost-effectiveness of early versus late PN in the context of the Australian public hospital system. Thus, our cost analysis in this RCT will provide a unique opportunity to gather data regarding the costs associated with the interventions from an Australian hospital perspective.

## Trial status

This study recruitment commenced on the 15th of June 2020. At the time of the safety interim analyses (after 20 and 40 study infants discharged from NICU), the DSMC advised the continuation of the study. As of the 14 November 2021, 54 patients have been recruited in this study. Recruitment of the last patient is expected by March 2022.

## Data Availability

Not applicable.

## Abbreviations

CPAP: Continuous positive airway pressure
CPI: Coordinating Principal Investigator
CRP: C-reactive protein
DSMC: Data safety monitoring committee
EFA: Essential fatty acids
EN: Enteral nutrition
HABSI: Healthcare-associated bloodstream infection
HFNP: Humidified high-flow nasal prong
HIE: Hypoxic-ischemic encephalopathy
HREC: Human research ethics committee
LCPUFA: Long-chain polyunsaturated fatty acids
MDI: Mental developmental index
NICU: Neonatal intensive care unit
NIPPV: Non-invasive positive-pressure ventilation
PCH: Perth Children’s Hospital
PMA: Postmenstrual age
PN: Parenteral nutrition
PPM: Power Performance Manager
PPHN: Persistent pulmonary hypertension of the newborn
RBC: Red blood cell
RCT: Randomized controlled trial
SNAPPE-II: Score for Neonatal Acute Physiology with Perinatal Extension-II
